# Identification of a Novel Porcine Teschovirus Subtype 19 within the Species *Teschovirus A*

**DOI:** 10.1155/2023/9977581

**Published:** 2023-12-11

**Authors:** Taotao Yang, Anqi Lin, Shuoyan Cui, Jing Chen, Hanhao Wan, Yingmei Lu, Pengcheng Li, Zhibang Zhang, Kai Li, Minhong Guo, Yanzhang Xu

**Affiliations:** ^1^College of Life Sciences and Resource Environment, Yichun University, Yichun 336000, Jiangxi, China; ^2^Jiangxi Sunshine Dairy Company Limited, Nanchang 330043, Jiangxi, China

## Abstract

Porcine teschovirus (PTV) is a member of the genus *Teschovirus* in the family *Picornaviridae*. Several novel PTVs in *Teschovirus* were identified in our previous study. To elucidate the taxonomic status of the species and subtypes, PTV-positive samples were inoculated into porcine kidney (PK-15) and swine testicular (ST) cells. A total of 58 PTV strains were successfully isolated and 21 different VP1 gene sequences were obtained. A phylogenetic analysis of the VP1 gene revealed that these PTV isolates comprised 11 genotypes, including 10 known genotypes (PTV 1–6, PTV 8–9, PTV 11, and Teschovirus B3), and a newly identified genotype. A nearly complete genome sequence of the novel PTV genotype isolate, PTV-SG2, was obtained. The homologies of the nucleotides and amino acids between PTV-SG2 and other PTVs were 69.7%–85.0% and 76.1%–90.4%, respectively. A phylogenetic analysis of the P1, polyprotein, and 3 CD genes revealed that PTV-SG2 belonged to a novel genotype within *Teschovirus A*, and a genetic divergence analysis of the novel PTV isolate further verified its taxonomic status. Based on the PTV naming convention, the novel PTV genotype identified in the present study was provisionally named PTV 19. Serum neutralization tests indicated that PTV 19 is a serotype-specific and completely different from previously reported PTV serotypes. Therefore, PTV 19 is a novel PTV serogroup. In conclusion, this study identified a PTV genotype and serotype within *Teschovirus A*.

## 1. Introduction

Various porcine teschovirus (PTV) subtypes (genotypes and serotypes) can cause diseases, including polioencephalomyelitis, reproductive and intestinal diseases, and pneumonia [1]. Severe teschovirus encephalomyelitis, also known as Teschen disease, is caused by the virulent strains of PTV 1 and PTV 11 and often leads to high-morbidity and mortality rates in pig populations, which can eventually result in significant economic losses [2, 3]. In most cases, pigs infected with other subtypes of PTV or attenuated strains of PTV 1 and PTV 11 exhibit only subclinical or mild clinical symptoms, including mild ataxia, hind limb progressive paralysis, mild polioencephalomyelitis, diarrhea, pneumonia, and myocarditis [1, 4–7]. These cases often have low-morbidity and mortality rates and mainly affect nursery and fattening pigs. In addition, PTV coinfections with other pathogens, such as PCV 2, may induce notable clinical symptoms, thereby increasing the risk of disease in pigs [8, 9].

PTV, a single positive-stranded RNA virus, is a member of the genus *Teschovirus* in the family *Picornaviridae* and includes serotypes PTV 1–12 [10]. Genotyping is widely used to classify the PTVs because viral isolates of the same serotype can share the same genotype. The genus *Teschovirus* was found to include the species *Teschovirus A* and *Teschovirus B* in the latest taxonomy and naming of PTV virus species and genotypes released by the International Committee on Taxonomy of Viruses (ICTV). *Teschovirus A* and *Teschovirus B* contain 14 genotypes (PTV 1–14) and 3 genotypes (Teschovirus B1–B3), respectively [11]. There are two other genotypes (PTV 15 and PTV 16) belonging to the interspecies recombinants of *Teschovirus A* and *Teschovirus B* [11]. PTV infections are prevalent in pigs in China, and almost all known PTV genotypes have been reported [12, 13]. Recently, novel strains that are different from the known PTV genotypes in genetic evolutionary relationships have been discovered [12–14]. However, only a short VP1 gene sequence has been obtained for most of the newly discovered PTV strains, which is insufficient to accurately establish the taxonomic statuses of the PTV species and genotypes. Furthermore, these novel PTV strains have not been previously identified using serological methods. Therefore, the multiple novel PTVs in the taxonomy of viral species and their subtypes remain unclear. In this study, the viral species and subtypes of a novel PTV strain were determined using genotyping and serotyping methods by obtaining their genomic sequences and isolates.

## 2. Materials and Methods

### 2.1. Virus Isolation

Sixty-seven PTV fecal samples (0.5 g) from healthy pigs, identified as positive in our previous study [12, 14], were freeze-thawed three times in 1 mL of PBS. The samples were then centrifuged for 20 min at 12,000× *g*. The supernatants were sterile filtration using 0.22-*μ*m filters (Pall Corp., Port Washington, NY, USA) and incubated on monolayers of porcine kidney (PK-15) and swine testicular (ST) cells. The cytopathic conditions were observed and recorded daily. Cultures were identified using the PTV quantitative real-time polymerase chain reaction (qRT-PCR) method developed in our previous study [15] after three passages.

### 2.2. Gene Amplification and Sequencing of PTV Isolates

The primer sets used for PTV VP1 gene and genome amplification were designed in our previous study [13]. A nested PCR was specially used to amplify the VP1 gene of PTV isolates. PCR amplification was first performed using external primers. Next, 1 *μ*L of the PCR product amplified using external primers was used as a template for the internal PCR reaction. The PCR reactions were both carried out using 2x PCR Phanta Max Master Mix (Vazyme, Nanjing, China) according to the manufacturer's instructions. The PCR products were analyzed using 0.8% agarose gel electrophoresis, and the remaining products were sequenced (BioSune, Shanghai, China).

### 2.3. Multiple Sequence Alignment and Genetic Evolution Analysis

Multiple alignments of PTV sequences were performed using the Mafft program [16]. Homology analyses between PTV strains were performed using the MegAlign program in DNAStar software [17]. Phylogenetic analyses were performed using MEGA 6.06 with the maximum-likelihood (ML) method based on Jones–Taylor–Thornton model with gamma-distributed rate heterogeneity and proportions of invariant sites (G + I) [18]. Support for the estimated phylogeny was evaluated using a bootstrap analysis with 1,000 replicates. For each amino acid (aa) sequence dataset, the genetic divergence between genotypes was calculated using the uncorrected p-distance model in MEGA 6.06 software [18].

### 2.4. Preparation of Hyperimmune Serum of the Novel PTV Isolate and Serum Neutralization Test

The novel PTV isolate was propagated with PK-15 cells. The harvested virus with complete cytopathic effects was freeze-thawed three times and centrifuged at 12,000× *g* for 30 min at 4°C. The fully emulsified immune antigen was prepared using 0.5 mL of vaccine adjuvant (ISA201VG, Seppic, Paris, France) with 0.5 mL of novel PTV suspension. Each of the two New Zealand rabbits used was subcutaneously injected with 1 mL of the vaccine in multiple places on the body. Booster immunization with a dose of 2 mL of vaccine was performed 4 and 8 weeks after the primary immunization. Serums were collected 10 days after the third immunization. Serum neutralization (SN) tests of the hyperimmune serum of the novel PTV isolate were performed using isolates of the novel PTV and other known PTV genotypes as antigens. The neutralizing antibody titers in the serum were measured using the microtiter method. First, the 50% tissue culture infectious dose (TCID_50_) of the PTV strains was determined. The serum to be tested were inactivated at 56°C for 30 min and diluted with twofold series. A volume of 50 *μ*L of serum with different dilutions was equally mixed with the PTV containing the virus titer at 200 TCID_50_/0.1 mL. The mixtures were incubated at 37°C for 1 hr and then with PK-15 or ST cell monolayers. Incubation at 37°C with 5% CO_2_ was continued for 4–5 days. Cytopathic conditions were observed and recorded daily and SN antibody titers were calculated with the Reed–Muench method. The SN antibody titer was expressed as the highest serum dilution showing the inhibition of cytopathic effects. A titer of 1 : 80 or more was considered as being specific to the corresponding PTV isolate [19].

## 3. Results and Discussion

**Figure 1:**
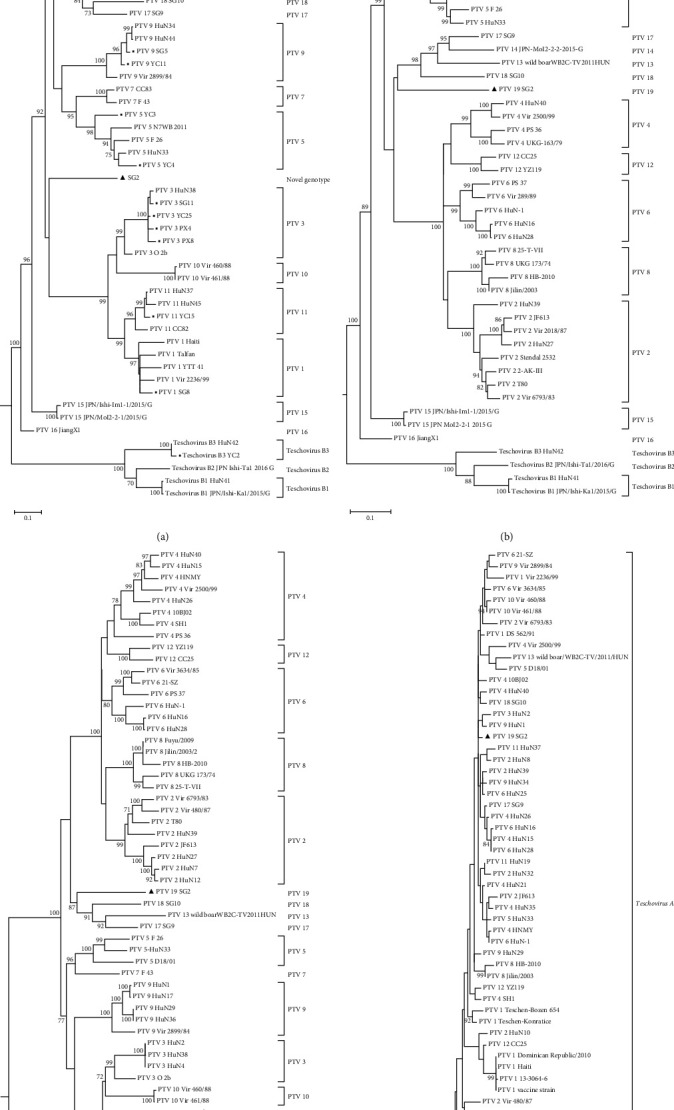
Evolutionary PTV relationships based on the amino acid sequences of the VP1 (a), P1 (b), polyprotein (c), and 3 CD (d) genes. The evolutionary history was inferred using the maximum-likelihood in the Jones–Taylor–Thornton model with gamma-distributed rates and proportions of invariant sites (G + I) in MEGA 6.06 software. The percentages of the replicates in which the associated virus clustered together in the bootstrap test (1,000 replicates) are shown next to the branches (only values >70% are shown) in each tree. There were 272, 878, 2,225, and 657 positions in the VP1, P1, polyprotein, and 3 CD datasets, respectively. The strains isolated in the present study are indicated by squares. The novel genotype isolate (PTV 19-SG2) is indicated by triangles.

We identified a total of 46 PTV strains from the PK-15 cells and 12 from the ST cells using qRT-PCR. A sequence alignment and homology analysis of VP1 in PTV isolates revealed 21 unique VP1 gene sequences. A phylogenetic analysis of the VP1 gene of the newly obtained PTV isolates showed that these isolates belonged to 11 genotypes, 10 of which are known PTV genotypes, including PTV 1–6, PTV 8–9, PTV 11, and Teschovirus B3 (Figure 1(a)). In addition, a novel PTV genotype was identified (Figure 1(a)).

The nearly complete genome of the novel PTV isolate was obtained by gene amplification, sequencing, and splicing and then submitted to GenBank (accession number OR364748). The genome sequence alignment of the novel PTV isolate with other known PTV reference strains (*Supplementary 1*) revealed that the novel isolate contained an open reading frame (ORF) of 6,627 nucleotides encoding a polyprotein of 2,209 amino acids. Comparisons between the ORF sequences of the novel isolate and those of other PTV reference strains revealed nucleotide and aa sequence identities of 69.7%–85.0% and 76.1%–90.4%, respectively (*Supplementary 2*).

To determine the taxonomic status of the novel PTV isolate (SG2), phylogenetic trees of the P1, polyprotein, and 3 CD genes of the isolate were constructed. Phylogenetic trees of both the P1 and polyprotein genes revealed that the SG2 isolate fell into a separate branch distinct from that of other known PTV genotype strains, indicating that the SG2 isolate belonged to a novel PTV genotype (Figures 1(b) and 1(c). According to the topological structure of the branches of the trees, the SG2 isolate was more closely related to the genotype strains of *Teschovirus A*. A 3 CD tree also demonstrated that the SG2 isolate was separated into a branch of *Teschovirus A*, indicating that the isolate belonged to *Teschovirus A* based on the taxonomic status of the species (Figure 1(d)). Therefore, the PTV-SG2 isolate represents a novel taxonomic genotype of *Teschovirus A*. According to the PTV naming convention, the novel PTV genotype was provisionally named PTV 19.

To further verify the taxonomic status of the novel PTV isolate (PTV 19-SG2), the aa genetic distances of the P1, polyprotein, and 2 C-3 CD genes between PTV 19-SG2 and PTV 1–14, 17–18 (*Teschovirus A*), and Teschovirus B1–B3 (*Teschovirus B*) were calculated. Interspecies recombinants (PTV 15 and PTV 16) between *Teschovirus A* and *Teschovirus B* were excluded. The average genetic distance of the P1 gene between PTV 19 and the *Teschovirus A* genotypes ranged from 0.212 ± 0.014 to 0.243 ± 0.014, whereas the average genetic distance between PTV 19 and the *Teschovirus B* genotypes was 0.353 ± 0.016 to 0.363 ± 0.016 (Table 1). The evolutionary distance of the polyprotein gene between PTV 19 and the *Teschovirus A* genotypes was 0.092 ± 0.006 to 0.118 ± 0.006, and the average genetic distance between PTV 19 and the *Teschovirus B* genotypes was 0.227 ± 0.009 to 0.232 ± 0.009 (Table 1). In addition, the average genetic distance of the 2 C-3 CD gene between PTV 19 and the *Teschovirus A* genotypes ranged from 0.011 ± 0.003 to 0.037 ± 0.006, whereas that between PTV 19 and the *Teschovirus B* genotypes was 0.144 ± 0.011 to 0.145 ± 0.011 (Table 1). Based on these findings and the species demarcation criteria of the genus *Teschovirus* published by the ICTV [11], PTV 19 belong to *Teschovirus A*. The P1 and VP1 genes are commonly used to PTV genotyping in the recent studies [12–14]. In the present study, the average genetic distances of the P1 gene between PTV 19 and the *Teschovirus A* genotypes (0.212 ± 0.014 to 0.243 ± 0.014) were markedly higher than those of within *Teschovirus A* genotypes (0.040 ± 0.006 to 0.092 ± 0.007) (*Supplementary 3*). The similar result also indicated by the genetic divergence analysis of VP1 gene (*Supplementary 4*). These results further verify PTV 19 is a novel genotype of *Teschovirus A*.

SN tests of the PTV 19 hyperimmune serum showed that the antibody titer against PTV 19 was 1 : 1,600, whereas the titers against the PTV isolates of other known genotypes identified in this study were all less than 1 : 10 (Table 2). Moreover, the seroneutralization antibody titers of other known PTV genotypes immune serums against PTV19 were also all less than 1 : 10 (Table 2). These findings indicate that PTV 19 is a serotype-specific and completely different from previously reported PTV serotypes. Therefore, PTV 19 is a new serogroup.

PTV was once classified in the genus *Enterovirus*, and the taxonomy of different viral species within the genus was based on the type of cytopathic formation, serological tests, and viral replication in different cell lines [20]. ICTV first proposed to create the genus *Teschovirus* in 1999, and *Porcine enterovirus* (PEV) type 1 was classified into this genus after being renamed PTV. In 2000, [21] made a more systematic classification of PEV based on its genome structure and evolutionary analysis. PEV 1–7 and PEV 11–13 were classified into the same viral species, *Porcine teschovirus*, which belongs to the genus *Teschovirus*. The original porcine enterovirus was serotyped according to a serum cross-neutralization test, and PEV 1–7 and PEV 11–13 were renamed PTV 1–7 and PTV 8–10, respectively. The study further identified a novel PTV serotype, PTV 11, and unexpectedly found that PTV strains of the same serotype have a close evolutionary relationship with P1. Thus, a phylogenetic tree for the P1 gene was constructed and proved to differentiate the PTV serotypes. Since then, other studies have found that the phylogenetic tree of partial structural protein genes, including the VP1 gene and “puff” region of the VP2 gene, can also distinguish PTV serotypes [22–26]. Thus, PTV serotypes identified by evolutionary analyses of gene sequences have been widely used in research. However, many novel strains that are different from PTV 1–11 in terms of gene phylogenetic relationships have been discovered with the increasingly widespread application of genome sequencing [12–14, 19, 23, 27]. Since then, genotyping methods have been widely applied for the classification of PTV.

Currently, the VP1 and P1 genes are the most commonly used genes for PTV genotyping. The VP1 gene sequence is short and can be easily obtained; therefore, it is often used in epidemiological investigations of PTV genotypes. Multiple novel PTV strains have recently been identified using VP1 genotyping [12–14]. In the present study, the genotype identification of PTV isolates was initially performed using VP1. A novel PTV genotype was identified using this method. However, the VP1 fragment was too short to accurately genotype the novel PTV. The ICTV recommends using the P1 gene to genotype novel PTVs. Therefore, we obtained a nearly complete genome sequence of the novel PTV isolate. An evolutionary analysis of P1 confirmed that this isolate belonged to a novel PTV genotype.

According to the criteria for species demarcation defined by the ICTV, members of a species of *Teschovirus* are less than 20% divergent in the polyprotein aa sequence, less than 30% divergent in the P1 aa sequence, less than 10% divergent in the 2C + 3CD aa sequence, and share a common genome organization and natural host range [11]. In this study, the average divergence of the polyprotein aa sequence was 10.9% between the novel PTV isolate (PTV-SG2) and genotype strains of *Teschovirus A* and 22.9% between the genotype strains of *Teschovirus B*. The average divergence of the P1 aa sequence between PTV-SG2 and the genotype strains of *Teschovirus A* was 22.8%, and that between the genotype strains of *Teschovirus B* was 35.7%. The average divergence of the 2C + 3CD aa sequence between PTV-SG2 and the genotype strains of *Teschovirus A* was 2.8%, and that between the genotype strains of *Teschovirus B* was 14.5% (data not shown). Therefore, PTV-SG2 is a member of *Teschovirus A*. This conclusion was confirmed by the phylogenetic relationship between PTV-SG2 and other PTV reference strains.

In addition to identifying the interspecies recombinants (PTV 15 and PTV 16) between *Teschovirus A* and *Teschovirus B*, recent studies have shown that the genus *Teschovirus* comprises 19 genotypes. *Teschovirus A* and *Teschovirus B* comprise 16 genotypes (PTV PTV 1–14, 17–18) and 3 genotypes (Teschovirus B1–B3), respectively [12–14, 19, 21, 23, 27]. PTV-SG2, which was isolated and identified in the present study, belongs to a novel PTV genotype within *Teschovirus A*. Based on the PTV naming convention, we provisionally named the genotype PTV 19. *Teschovirus A* comprises 17 genotypes now.

Although genotyping is widely used for classifying PTVs, serotyping is the most direct method for identifying virus antigenicity and immunogenicity. To date, 12 serotypes of PTV have been identified, namely, PTV 1–12 [19, 21]. In the present study, the hyperimmune serum of PTV 19 was prepared for serotype identification. SN tests showed that the immune serum produced a high neutralizing antibody titer against PTV 19 but had low titers against PTV strains of other known genotypes that have been isolated in our laboratory. This result suggests PTV 19 is a novel PTV serogroup. However, the neutralizing antibody reactions of the PTV 19 immune sera against PTV 7, PTV 10, and PTV 12 remain unknown because these serotype strains have not yet been successfully isolated. These factors should be verified in the future studies.

## 4. Conclusions

In the present study, 58 PTV strains were successfully isolated, and 57 PTV strains were identified as known PTV genotypes, including PTV 1–6, PTV 8–9, PTV 11, and *Teschovirus* B3. Additionally, PTV-SG2 was identified. The evolutionary analysis proved that PTV-SG2 belongs to a novel *Teschovirus A* genotype. Further serotyping revealed that PTV-SG2 is also a novel PTV serogroup. These findings contribute to enriching the knowledge of PTV taxonomy.

## Figures and Tables

**Table 1:** Estimates of evolutionary divergence between PTV 19 and the other known PTV genotypes within *teschovirus A* and *teschovirus B* based on the aa sequences of P1, polyprotein and 2 C–3 CD genes.

Species	Genotypes^a^	Genes		
		P1	Polyprotein	2 C–3 CD
*Teschovirus A*	PTV 1	0.220 ± 0.014	0.109 ± 0.006	0.034 ± 0.005
	PTV 2	0.233 ± 0.014	0.109 ± 0.005	0.027 ± 0.004
	PTV 3	0.227 ± 0.013	0.107 ± 0.006	0.029 ± 0.005
	PTV 4	0.243 ± 0.014	0.113 ± 0.005	0.023 ± 0.003
	PTV 5	0.217 ± 0.013	0.106 ± 0.006	0.031 ± 0.004
	PTV 6	0.218 ± 0.014	0.104 ± 0.005	0.024 ± 0.004
	PTV 7	0.225 ± 0.013	0.108 ± 0.006	0.033 ± 0.006
	PTV 8	0.233 ± 0.014	0.112 ± 0.006	0.033 ± 0.005
	PTV 9	0.243 ± 0.014	0.109 ± 0.006	0.021 ± 0.004
	PTV 10	0.237 ± 0.015	0.112 ± 0.007	0.027 ± 0.005
	PTV 11	0.222 ± 0.014	0.107 ± 0.006	0.028 ± 0.004
	PTV 12	0.230 ± 0.015	0.112 ± 0.006	0.026 ± 0.004
	PTV 13	0.234 ± 0.014	0.118 ± 0.006	0.037 ± 0.006
	PTV 14	0.227 ± 0.015	NA^b^	NA
	PTV 17	0.212 ± 0.014	0.092 ± 0.006	0.011 ± 0.003
	PTV 18	0.230 ± 0.014	0.106 ± 0.006	0.022 ± 0.005

*Teschovirus B*	TV-B1	0.353 ± 0.016	0.227 ± 0.009	0.145 ± 0.011
	TV-B2	0.360 ± 0.016	0.231 ± 0.009	0.145 ± 0.011
	TV-B3	0.363 ± 0.016	0.232 ± 0.009	0.144 ± 0.011

^a^The interspecies recombinants (PTV 15 and PTV 16) between *Teschovirus A* and *Teschovirus B* have been excluded. ^b^Not applicable, genome sequence of PTV 14 was incomplete.

**Table 2:** Seroneutralization titers against porcine teschovirus strains available in the laboratory using specific hyperimmune antisera.

Antigen^a^	Strain	GenBank number	Antiserum (AS)	Seroneutralization titer
PTV 1	SG8	MN162706	AS.PTV 19	<1 : 10
PTV 2	YC24	OR364743	AS.PTV 19	<1 : 10
PTV 3	YC25	OR364744	AS.PTV 19	<1 : 10
PTV 4	SG3	MN162701	AS.PTV 19	<1 : 10
PTV 5	YC3	MN094611	AS.PTV 19	<1 : 10
PTV 6	YC26	OR364745	AS.PTV 19	<1 : 10
PTV 8	YC18	MN094626	AS.PTV 19	<1 : 10
PTV 9	SG5	MN162703	AS.PTV 19	<1 : 10
PTV 11	YC15	MN094623	AS.PTV 19	<1 : 10
Teschovirus B3	YC2	MN094610	AS.PTV 19	<1 : 10

PTV 19	SG2	OR364748	AS.PTV 19	1 : 1600
			AS.PTV 1	<1 : 10
			AS.PTV 2	<1 : 10
			AS.PTV 3	<1 : 10
			AS.PTV 4	<1 : 10
			AS.PTV 5	<1 : 10
			AS.PTV 6	<1 : 10
			AS.PTV 8	<1 : 10
			AS.PTV 9	<1 : 10
			AS.PTV 11	<1 : 10
			AS.Teschovirus B3	<1 : 10

^a^The antigens and antisera of PTV 7, PTV 10, and PTV 12 are not tested by the reason of these three serotype strains have not successfully isolated in our laboratory.

## Data Availability

Sequence of the novel PTV subtype (PTV 19) was deposited in the GenBank (accession number OR364748).

## Supplementary Materials

Supplementary 1 Porcine teschovirus reference strains used in the study.

Supplementary 2 Homology of polyprotein gene between the novel PTV isolate and other PTV genotypes.

Supplementary 3 The estimates of evolutionary divergence over sequence pairs within and between PTV genotypes identified by the neighbor-joining method, which include *P1* sequence of 63 PTV reference strains.

Supplementary 4 The estimates of evolutionary divergence over sequence pairs within and between PTV genotypes identified by the neighbor-joining method, which include VP1 sequence of 67 PTV reference strains.
