# Supplementary material for: Identification of a Novel Porcine Teschovirus Subtype 19 within the Species Teschovirus A
Source: Transbound Emerg Dis. 2023 Dec 11;2023:9977581. doi: 10.1155/2023/9977581 (PMC12017212; doi:10.1155/2023/9977581)
Supplement: Supplementary 1 — Porcine teschovirus reference strains used in the study. [file 9977581.f1.docx]

Supplementary Table 1 Porcine teschovirus reference strains used in the study.

| Virus isolate | Genotype | Collection date | GenBank accession No. | Geographic origin | Sequence length |
| --- | --- | --- | --- | --- | --- |
| DS 562/91 | 1 | 1991 | AF296100 | Germany | 7008 |
| Teschen-Bozen 654 | 1 | 1929 | AF231767 | Italy | 7109 |
| Teschen-Konratice | 1 | 1929 | AF231768 | Czech Republic | 7013 |
| Talfan | 1 | 1957 | AF231769 | UK | 7108 |
| swine/CH/IMH/03 | 1 | 2003 | DQ355222 | China | 6860 |
| Vir 2236/99 | 1 | 1999 | AF296102 | Germany | 7009 |
| D_61/96 | 1 | 1996 | AY392535 | Germany | 3685 |
| DS_1520/93 | 1 | 1993 | AY392532 | Germany | 3744 |
| Sek_549/98 | 1 | 1998 | AF296101 | Germany | 3434 |
| Teschen-Tirol | 1 | 1929 | AF296097 | Czech Republic | 3395 |
| Dominican Republic/2010 | 1 | 2010 | JQ808131 | Dominican Republic | 6921 |
| F65 | 1 | 1967 | AJ011380 | UK | 7117 |
| vaccine strain | 1 | - | KJ152768 | USA | 7110 |
| 13-3064-6 | 1 | 2013 | KC667563 | Dominican Republic | 7155 |
| Haiti | 1 | 2008 | GQ914053 | Haiti | 7155 |
| Vir 6793/83 | 2 | 1983 | AF296108 | Germany | 7017 |
| 2-AK-III | 2 | - | AY392542 | Germany | 3583 |
| Stendal_2532 | 2 | - | AY392537 | Germany | 3687 |
| Vir 2018/87 | 2 | 1987 | GQ293229 | Germany | 4060 |
| Vir 480/87 | 2 | 1987 | AF296109 | Germany | 7019 |
| T80 | 2 | 1958 | AF296087 | UK | 7017 |
| CC76 | 2 | 2005 | JF724034 | Spain | 792 |
| JF613 | 2 | 2009 | GU446660 | China | 7051 |
| HuN7 | 2 | 2016 | MF170911 | China | 6803 |
| HuN8 | 2 | 2016 | MF170912 | China | 6857 |
| HuN10 | 2 | 2016 | MF170914 | China | 6857 |
| HuN12 | 2 | 2016 | MF170916 | China | 6790 |
| HuN27 | 2 | 2016 | MF170931 | China | 6842 |
| HuN32 | 2 | 2017 | MF170936 | China | 6804 |
| HuN39 | 2 | 2017 | MF170943 | China | 6805 |
| O 2b | 3 | 1965 | AF296088 | USA | 7012 |
| 1-AA-VI | 3 | - | AY392540 | Germany | 3782 |
| HuN2 | 3 | 2016 | MF170906 | China | 6850 |
| HuN4 | 3 | 2016 | MF170908 | China | 6868 |
| HuN38 | 3 | 2016 | MF170942 | China | 6862 |
| Vir 1925/02 | 3 | 2002 | GQ293230 | Germany | 3709 |
| Vir 3764/86 | 4 | 1986 | AF296112 | Germany | 7015 |
| Vir 2500/99 | 4 | 1999 | AF296113 | Germany | 7015 |
| SH1 | 4 | 2010 | KJ881010 | China | 7097 |
| 10BJ02 | 4 | 2010 | JQ975417 | China | 6972 |
| PS 36 | 4 | 1965 | AF296089 | USA | 7014 |
| UKG-163/79 | 4 | 1979 | GQ293232 | UK | 4209 |
| HNMY | 4 | - | KX686489 | China | 7192 |
| HuN15 | 4 | 2016 | MF170919 | China | 6864 |
| HuN21 | 4 | 2016 | MF170925 | China | 6865 |
| HuN26 | 4 | 2016 | MF170930 | China | 6863 |
| HuN35 | 4 | 2017 | MF170939 | China | 6796 |
| HuN40 | 4 | 2015 | MF170944 | China | 6803 |
| D18/01 | 5 | 2001 | GQ293233 | Germany | 7117 |
| Vir 1806/89 | 5 | 1989 | AF296114 | Germany | 3254 |
| F 26 | 5 | 1958 | AF296090 | USA | 7008 |
| BL7792 | 5 | - | GQ293236 | Germany | 3631 |
| N7WB_2011 | 5 | 2011 | JX069833 | Spain | 783 |
| HuN33 | 5 | 2014 | MF170937 | China | 6796 |
| 21-SZ | 6 | 1963 | AF296117 | Hungary | 7018 |
| Vir 3634/85 | 6 | 1985 | AF296115 | Germany | 7017 |
| Vir 289/89 | 6 | 1989 | AF296116 | Germany | 3784 |
| CC51 | 6 | 2005 | JF724021 | Spain | 792 |
| PS 37 | 6 | 1965 | AF296091 | USA | 7018 |
| HuN-1 | 6 | 2013 | KU297677 | China | 7098 |
| HuN16 | 6 | 2016 | MF170920 | China | 6845 |
| HuN25 | 6 | 2016 | MF170929 | China | 6868 |
| HuN28 | 6 | 2016 | MF170932 | China | 6845 |
| CC83 | 7 | 2009 | JF724041 | Spain | 786 |
| F 43 | 7 | 1958 | AF296092 | Germany | 7014 |
| WR-2 | 7 | 1957 | GQ293237 | USA | 3599 |
| 25-T-VII | 8 | 1963 | AF296118 | Hungary | 7020 |
| UKG 173/74 | 8 | 1974 | AF296093 | UK | 7017 |
| CC30 | 8 | 2005 | JF724006 | Spain | 792 |
| Jilin/2003 | 8 | 2003 | GQ293092 | China | 7106 |
| HB-2010 | 8 | 2010 | JQ664746 | China | 7090 |
| Fuyu/2009 | 8 | 2009 | HQ020378 | China | 7066 |
| Jilin/2003/2 | 8 | 2003 | JN710381 | China | 7126 |
| Vir 2899/84 | 9 | 1984 | AF296094 | Germany | 7006 |
| HuN1 | 9 | 2016 | MF170905 | China | 6853 |
| HuN17 | 9 | 2016 | MF170921 | China | 6791 |
| HuN29 | 9 | 2016 | MF170932 | China | 6845 |
| HuN34 | 9 | 2017 | MF170938 | China | 6793 |
| HuN36 | 9 | 2016 | MF170940 | China | 6790 |
| HuN44 | 9 | 2017 | MG875518 | China | 783 |
| Vir 461/88 | 10 | 1988 | AF296119 | Germany | 7009 |
| Vir 460/88 | 10 | 1988 | AF296095 | Germany | 7009 |
| Dresden | 11 | 1965 | AF296096 | Germany | 7111 |
| RD_181/01 | 11 | 2001 | AY392536 | Germany | 3284 |
| 1008/88 | 11 | 1988 | AY392550 | Germany | 3894 |
| HuN19 | 11 | 2016 | MF170923 | China | 6875 |
| CC82 | 11 | 2009 | JF724040 | Spain | 789 |
| HuN45 | 11 | 2017 | MG875519 | China | 789 |
| HuN37 | 11 | 2016 | MF170941 | China | 6856 |
| CC25 | 12 | 2006 | JN859128 | Spain | 6952 |
| YZ119 | 12 | 2012 | KR018369 | China | 7075 |
| Wild boar/WB2C-TV/2011/HUN | 13 | 2011 | JQ429405 | Hungary | 7123 |
| JPN/MoI2-2-2/2015/G | 14 | 2015 | LC386160 | Japan | 3342 |
| JPN/Ishi-Im1-1/2015/G | 15 | 2015 | LC386158 | Japan | 6820 |
| JPN/MoI2-2-1/2015/G | 15 | 2015 | LC386159 | Japan | 6445 |
| JiangX1 | 16 | 2019 | MN094632 | China | 6799 |
| SG9 | 17 | 2019 | MN162707 | China | 6773 |
| SG10 | 18 | 2019 | MN162708 | China | 6776 |
| HuN41 | B1 | 2017 | MG875515 | China | 6931 |
| JPN/Ishi-Ka1/2015/G | B1 | 2015 | LC386154 | Japan | 7138 |
| JPN/Ishi-Ta1/2016/G | B2 | 2016 | LC386156 | Japan | 7163 |
| HuN42 | B3 | 2017 | MG875516 | China | 6922 |

-, Not identified.
