# Supplementary material for: Identification of a Novel Porcine Teschovirus Subtype 19 within the Species Teschovirus A
Source: Transbound Emerg Dis. 2023 Dec 11;2023:9977581. doi: 10.1155/2023/9977581 (PMC12017212; doi:10.1155/2023/9977581)
Supplement: Supplementary 2 — Homology of polyprotein gene between the novel PTV isolate and other PTV genotypes. [file 9977581.f2.docx]

Supplementary Table 2 Homology of polyprotein gene between the novel PTV isolate and other PTV genotypes

| Genotypes^a^ | Nucleotide sequence identity | Amino acid sequence identity |
| --- | --- | --- |
| PTV 1 | 80.2%-81.3% | 88.0%-89.1% |
| PTV 2 | 80.9%-83.7% | 88.0%-89.5% |
| PTV 3 | 80.3%-82.7% | 88.5%-89.0% |
| PTV 4 | 80.2%-83.7% | 87.5%-89.0% |
| PTV 5 | 80.7%-82.8% | 88.6%-89.5% |
| PTV 6 | 81.3%-84.5% | 89.0%-90.2% |
| PTV 7 | 81.1% | 88.8% |
| PTV 8 | 80.5%-82.2% | 87.2%-88.9% |
| PTV 9 | 81.0%-83.2% | 88.5%-88.9% |
| PTV 10 | 80.4%-80.6% | 88.4%-88.5% |
| PTV 11 | 80.2%-83.1% | 87.8%-89.5% |
| PTV 12 | 81.1%-82.1% | 87.4%-88.4% |
| PTV 13 | 81.1% | 87.6% |
| PTV 15 | 73.8% | 81.4% |
| PTV 16 | 71.9% | 79.7% |
| PTV 17 | 85.0% | 90.4% |
| PTV 18 | 82.0% | 88.9% |
| Teschovirus B1 | 70.6%-70.7% | 76.4%-76.8% |
| Teschovirus B2 | 69.7% | 76.1% |
| Teschovirus B3 | 70.1% | 76.3% |

^a^ PTV 14 was not included for its incomplete genome sequence.
