# Supplementary material for: Identification of a Novel Porcine Teschovirus Subtype 19 within the Species Teschovirus A
Source: Transbound Emerg Dis. 2023 Dec 11;2023:9977581. doi: 10.1155/2023/9977581 (PMC12017212; doi:10.1155/2023/9977581)
Supplement: Supplementary 3 — The estimates of evolutionary divergence over sequence pairs within and between PTV genotypes identified by the neighbor-joining method, which include P1 sequence of 63 PTV reference strains. [file 9977581.f3.docx]

Supplementary Table 3 The estimates of evolutionary divergence over sequence pairs within and between PTV genotypes identified by the neighbor-joining method, which include *P1* sequence of 63 PTV reference strains.

| Genetic distance within PTV genotypes (mean ± SE ) | Average genetic distance between PTV genotypes (mean ± SE ) | | | | | | | | | | | | | | | | | | | | |
| --- | --- | --- | --- | --- | --- | --- | --- | --- | --- | --- | --- | --- | --- | --- | --- | --- | --- | --- | --- | --- | --- |
|  | Genotype^a^ | PTV 1 | PTV 2 | PTV 3 | PTV 4 | PTV 5 | PTV 6 | PTV 7 | PTV 8 | PTV 9 | PTV 10 | PTV 11 | PTV 12 | PTV 13 | PTV 14 | PTV 17 | PTV 18 | PTV 19 | TV-B1 | TV-B2 | TV-B3 |
| 0.061±0.005 | PTV 1 |  | - | - | - | - | - | - | - | - | - | - | - | - | - | - | - | - | - | - | - |
| 0.074±0.006 | PTV 2 | 0.230±0.013 |  | - | - | - | - | - | - | - | - | - | - | - | - | - | - | - | - | - | - |
| 0.082±0.007 | PTV 3 | 0.145±0.010 | 0.228±0.013 |  | - | - | - | - | - | - | - | - | - | - | - | - | - | - | - | - | - |
| 0.092±0.007 | PTV 4 | 0.236±0.013 | 0.151±0.010 | 0.229±0.013 |  | - | - | - | - | - | - | - | - | - | - | - | - | - | - | - | - |
| 0.090±0.006 | PTV 5 | 0.205±0.012 | 0.222±0.013 | 0.188±0.011 | 0.211±0.012 |  | - | - | - | - | - | - | - | - | - | - | - | - | - | - | - |
| 0.074±0.007 | PTV 6 | 0.224±0.012 | 0.142±0.010 | 0.220±0.012 | 0.138±0.010 | 0.205±0.012 |  | - | - | - | - | - | - | - | - | - | - | - | - | - | - |
| 0.040±0.006 | PTV 7 | 0.213±0.012 | 0.225±0.013 | 0.187±0.011 | 0.225±0.013 | 0.170±0.011 | 0.212±0.013 |  | - | - | - | - | - | - | - | - | - | - | - | - | - |
| 0.057±0.006 | PTV 8 | 0.233±0.013 | 0.147±0.010 | 0.237±0.013 | 0.151±0.011 | 0.221±0.013 | 0.147±0.010 | 0.231±0.013 |  | - | - | - | - | - | - | - | - | - | - | - | - |
| 0.048±0.005 | PTV 9 | 0.213±0.012 | 0.225±0.014 | 0.201±0.012 | 0.224±0.013 | 0.195±0.013 | 0.211±0.012 | 0.193±0.013 | 0.234±0.014 |  | - | - | - | - | - | - | - | - | - | - | - |
| 0.006±0.002 | PTV 10 | 0.170±0.011 | 0.244±0.013 | 0.149±0.011 | 0.247±0.013 | 0.210±0.012 | 0.232±0.013 | 0.212±0.012 | 0.243±0.014 | 0.216±0.013 |  | - | - | - | - | - | - | - | - | - | - |
| 0.068±0.006 | PTV 11 | 0.102±0.008 | 0.224±0.013 | 0.150±0.10 | 0.227±0.013 | 0.198±0.012 | 0.219±0.012 | 0.206±0.012 | 0.225±0.013 | 0.215±0.013 | 0.170±0.011 |  | - | - | - | - | - | - | - | - | - |
| 0.058±0.008 | PTV 12 | 0.223±0.013 | 0.142±0.011 | 0.228±0.013 | 0.139±0.010 | 0.213±0.013 | 0.137±0.010 | 0.226±0.014 | 0.151±0.011 | 0.220±0.013 | 0.241±0.014 | 0.216±0.013 |  | - | - | - | - | - | - | - | - |
| NA* | PTV 13 | 0.230±0.013 | 0.233±0..014 | 0.226±0.013 | 0.230±0.013 | 0.223±0.013 | 0.231±0.013 | 0.220±0.014 | 0.238±0.014 | 0.216±0.014 | 0.242±0.014 | 0.222±0.013 | 0.228±0.013 |  | - | - | - | - | - | - | - |
| NA* | PTV 14 | 0.228±0.013 | 0.227±0.013 | 0.218±0.013 | 0.227±0.013 | 0.211±0.013 | 0.219±0.013 | 0.219±0.013 | 0.230±0.014 | 0.212±0.013 | 0.238±0.014 | 0.222±0.013 | 0.222±0.014 | 0.169±0.012 |  | - | - | - | - | - | - |
| NA* | PTV 17 | 0.211±0.013 | 0.221±0.014 | 0.206±0.013 | 0.219±0.013 | 0.205±0.013 | 0.218±0.014 | 0.214±0.013 | 0.223±0.014 | 0.212±0.013 | 0.223±0.013 | 0.204±0.013 | 0.218±0.014 | 0.160±0.013 | 0.130±0.011 |  | - | - | - | - | - |
| NA* | PTV 18 | 0.241±0.014 | 0.235±0.013 | 0.237±0.014 | 0.224±0.013 | 0.221±0.013 | 0.212±0.013 | 0.218±0.013 | 0.233±0.013 | 0.229±0.014 | 0.254±0.014 | 0.230±0.014 | 0.233±0.014 | 0.206±0.014 | 0.199±0.013 | 0.180±0.013 |  | - | - | - | - |
| NA* | PTV 19 | 0.220±0.014 | 0.233±0.014 | 0.227±0.013 | 0.243±0.014 | 0.217±0.013 | 0.218±0.014 | 0.225±0.013 | 0.233±0.014 | 0.243±0.014 | 0.237±0.015 | 0.222±0.014 | 0.230±0.015 | 0.234±0.014 | 0.227±0.015 | 0.212±0.014 | 0.230±0.014 |  | - | - | - |
| 0.012±0.004 | TV-B1 | 0.342±0.016 | 0.338±0.015 | 0.353±0.015 | 0.350±0.015 | 0.340±0.015 | 0.351±0.015 | 0.348±0.016 | 0.364±0.015 | 0.345±0.015 | 0.350±0.016 | 0.337±0.016 | 0.351±0.015 | 0.353±0.016 | 0.344±0.016 | 0.353±0.016 | 0.349±0.016 | 0.353±0.016 |  | - | - |
| NA* | TV-B2 | 0.353±0.016 | 0.336±0.015 | 0.348±0.015 | 0.347±0.015 | 0.338±0.016 | 0.344±0.016 | 0.343±0.016 | 0.363±0.016 | 0.341±0.015 | 0.351±0.016 | 0.350±0.016 | 0.355±0.015 | 0.347±0.016 | 0.331±0.016 | 0.344±0.016 | 0.350±0.016 | 0.360±0.016 | 0.125±00.011 |  | - |
| NA* | TV-B3 | 0.357±0.016 | 0.036±0.015 | 0.359±0.016 | 0.352±0.015 | 0.344±0.016 | 0.352±0.016 | 0.356±0.016 | 0.362±0.015 | 0.356±0.016 | 0.352±0.016 | 0.352±0.016 | 0.361±0.016 | 0.366±0.016 | 0.350±0.016 | 0.362±0.016 | 0.363±0.017 | 0.363±0.016 | 0.156±0.012 | 0.148±0.012 |  |

The numbers of amino acid differences per site from averaging over all sequence pairs between and within PTV genotypes are shown. Standard error (SE) estimates are shown and were obtained by a bootstrap procedure (1000 replicates). The analyses involved 63 amino acid sequences were conducted using the p-distance model. All positions containing gaps and missing data were eliminated. There were a total of 845 positions in the final dataset. Evolutionary analyses were conducted in MEGA6.

**^a^** The interspecies recombinants (PTV 15 and PTV 16) between *Teschovirus A* and *Teschovirus B* have been excluded.

* Not applicable, only one sequence deposited in GenBank.
